# Radiomics nomogram for prediction of glypican-3 positive hepatocellular carcinoma based on hepatobiliary phase imaging

**DOI:** 10.3389/fonc.2023.1209814

**Published:** 2023-09-29

**Authors:** Ning Zhang, Minghui Wu, Yiran Zhou, Changjiang Yu, Dandan Shi, Cong Wang, Miaohui Gao, Yuanyuan Lv, Shaocheng Zhu

**Affiliations:** ^1^ Henan Provincial People’s Hospital, Xinxiang Medical University, Xinxiang, China; ^2^ Department of Medical Imaging, Henan Provincial People’s Hospital, Zhengzhou, China

**Keywords:** Gd-EOB-DTPA, hepatocellular carcinoma, glypican-3, radiomics, hepatobiliary phase, magnetic resonance imaging

## Abstract

**Introduction:**

The hepatobiliary-specific phase can help in early detection of changes in lesion tissue density, internal structure, and microcirculatory perfusion at the microscopic level and has important clinical value in hepatocellular carcinoma (HCC). Therefore, this study aimed to construct a preoperative nomogram for predicting the positive expression of glypican-3 (GPC3) based on gadoxetic acid-enhanced (Gd-EOB-DTPA) MRI hepatobiliary phase (HBP) radiomics, imaging and clinical feature.

**Methods:**

We retrospectively included 137 patients with HCC who underwent Gd-EOB-DTPA-enhanced MRI and subsequent liver resection or puncture biopsy at our hospital from January 2017 to December 2021 as training cohort. Subsequently collected from January 2022 to June 2023 as a validation cohort of 49 patients, Radiomic features were extracted from the entire tumor region during the HBP using 3D Slicer software and screened using a t-test and least absolute shrinkage selection operator algorithm (LASSO). Then, these features were used to construct a radiomics score (Radscore) for each patient, which was combined with clinical factors and imaging features of the HBP to construct a logistic regression model and subsequent nomogram model. The clinicoradiologic, radiomics and nomogram models performance was assessed by the area under the curve (AUC), calibration, and decision curve analysis (DCA). In the validation cohort,the nomogram performance was assessed by the area under the curve (AUC).

**Results:**

In the training cohort, a total of 1688 radiomics features were extracted from each patient. Next, radiomics with ICCs<0.75 were excluded, 1587 features were judged as stable using intra- and inter-class correlation coefficients (ICCs), 26 features were subsequently screened using the t-test, and 11 radiomics features were finally screened using LASSO. The nomogram combining Radscore, age, serum alpha-fetoprotein (AFP) >400ng/mL, and non-smooth tumor margin (AUC=0.888, sensitivity 77.7%, specificity 91.2%) was superior to the radiomics (AUC=0.822, sensitivity 81.6%, specificity 70.6%) and clinicoradiologic (AUC=0.746, sensitivity 76.7%, specificity 64.7%) models, with good consistency in calibration curves. DCA also showed that the nomogram had the highest net clinical benefit for predicting GPC3 expression.In the validation cohort, the ROC curve results showed predicted GPC3-positive expression nomogram model AUC, sensitivity, and specificity of 0.800, 58.5%, and 100.0%, respectively.

**Conclusion:**

HBP radiomics features are closely associated with GPC3-positive expression, and combined clinicoradiologic factors and radiomics features nomogram may provide an effective way to non-invasively and individually screen patients with GPC3-positive HCC.

## 1 Introduction

Liver cancer is a global health challenge and its incidence is increasing worldwide, especially in China, where it is the second leading cause of cancer-related death (1). More than 80% of patients with liver cancer are clinically diagnosed at the moderate to advanced stage, with a poor prognosis despite various treatments. Notably, the World Health Organization estimates that more than 1 million individuals will die from liver cancer by 2030 (2). Hepatocellular carcinoma (HCC) accounting for 75–85% of liver cancer and is a major type of liver cancer with high histological and molecular heterogeneity.

HCC-expressing stem cell markers are a new subtype that has been identified with different developmental pathways and unique morphological and immunohistochemical features. Glypican-3 (GPC3) is one such HCC stem cell marker that is a specific antigenic protein typically expressed in the liver during fetal development, but not in healthy adults or fatty liver disease, cirrhosis, and hepatitis. However, GPC3 is highly expressed in 80% of HCC tissues and is closely associated with elevated serum alpha-fetoprotein (AFP) levels (3–5), making it an ideal early diagnostic marker and therapeutic target for HCC. Evidence shows that patients with high GPC3 expression experienced higher recurrence and shorter survival rates than those with low GPC3 expression (6–8). Therefore, preoperative evaluation of GPC3 expression is important to improve patient outcomes and treatment strategies.

Recently, the development of medical imaging technology has facilitated preoperative non-invasive GPC3 assessment. Conventional methods include serological examination and liver biopsy; however these are limited by low sensitivity and high invasiveness, respectively. Similarly, traditional medical imaging modalities can only show simple features of lesions and organs, such as morphology, size, and mode of enhancement (9). However, radiomics, an emerging technology in the field of diagnostic imaging for high-throughput extraction of biological features, can transform medical images into quantitative data to enable model creation from large-scale datasets using computer algorithms. Furthermore, radiomic profiling has significant advantages and broad applicability in the diagnosis, prognosis prediction, and selection of suitable treatment options for tumors, which can aid in the early and non-invasive assessment of GPC3 expression in patients with HCC (10). In addition, gadolinium-ethoxybenzyl-diethylenetriamine pentaacetic acid (Gd-EOB-DTPA)-enhanced magnetic resonance imaging (MRI) can be used to evaluate quantitative and qualitative intratumoral and peritumoral imaging features during tumor development or heterogeneous and hemodynamic patterns in very early-stage HCC. Moreover, clinicoradiologic models based on Gd-EOB-DTPA-enhanced MRI features can aid qualitative patient diagnosis, efficacy evaluation, and postoperative recurrence prediction (11, 12).

Various studies have demonstrated a correlation between GPC3 expression and MRI radiomics features, histogram analysis, iterative decomposition of water and fat with echo asymmetry and least squares estimation quantification sequence (IDEAL-IQ) sequences, and liver imaging reporting and data system (LI-RADs) signatures (13–17). However, only one study reported the potential advantages of Gd-EOB-DTPA-enhanced MRI radiomics in identifying GPC3-positive HCC (13) and was based on multiple sequences with limited extracted features of the hepatobiliary phase (HBP). Changes in lesion tissue density, internal structure, and microcirculatory perfusion can be detected during the hepatobiliary-specific phase at the microscopic level early on and has important clinical value. Therefore, this study aimed to construct a nomogram model containing HBP information, including higher throughput radiomics features and qualitative and quantitative parameters of traditional medical imaging, and to explore the clinical application of the nomogram to predict GPC3 expression.

## 2 Materials and methods

### 2.1 Study population

**Figure 1:**
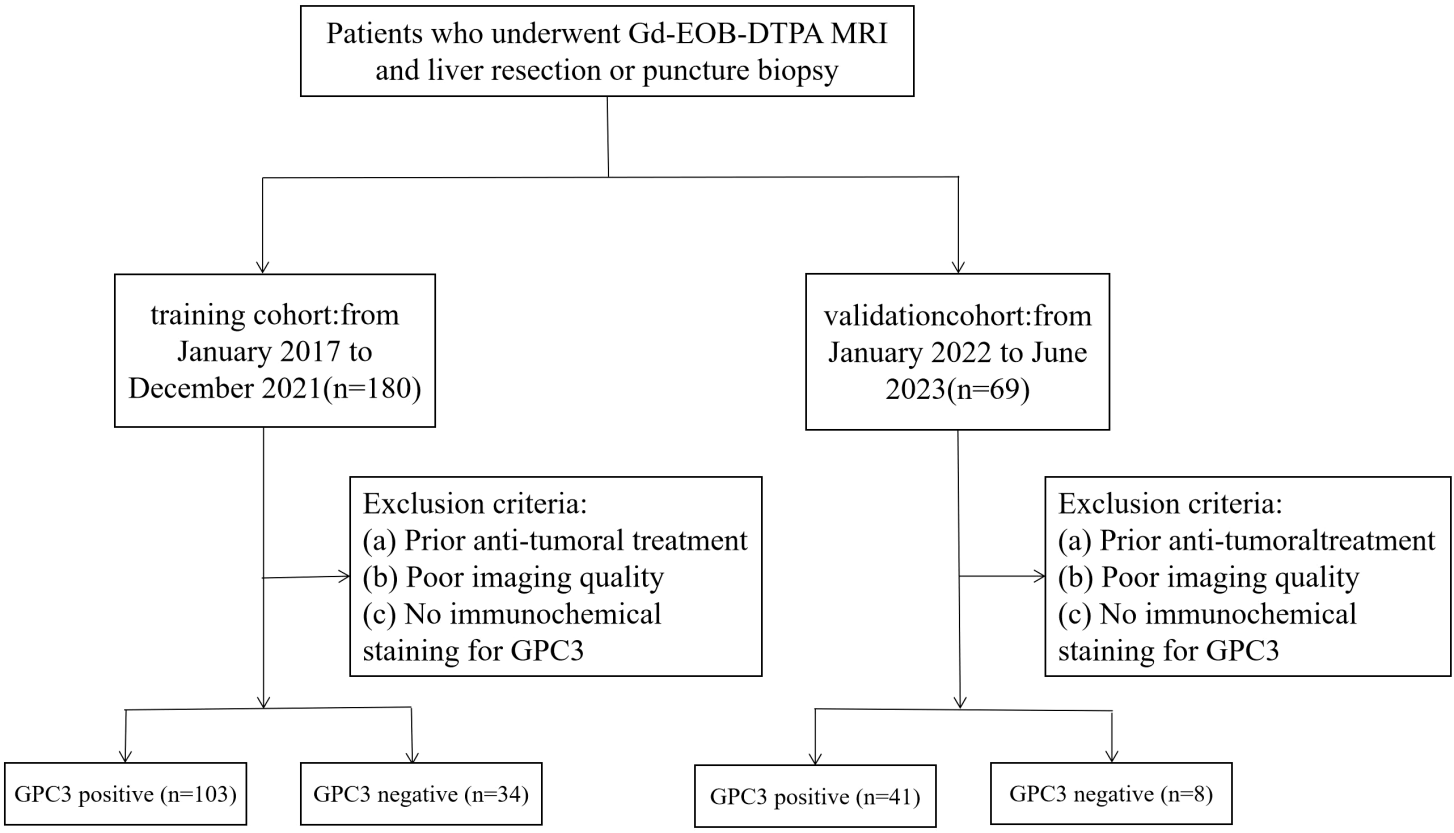
Flowchart of patient enrollment in the study.

This study retrospectively analyzed the data of 137 patients from January 2017 to December 2021 in Henan Provincial People’s Hospital as a training cohort, consisting of 103 patients in the GPC3 expression positive group and 34 patients in the GPC3 expression negative group. Subsequently collected from January 2022 to June 2023 as a validation cohort of 49 patients, of which 41 were GPC3 positive and 8 were negative, the inclusion criteria were as follows: (a) patients with HCC, confirmed by postoperative pathology report; (b) the use of Gd-EOB-DTPA-enhanced MRI, within one month before surgery; (c) complete GPC3 immunohistochemical staining; and (d) complete medical history. The exclusion criteria were as follows: (a) patients who received treatment for HCC before the MRI examination, such as transarterial chemoembolization, radiofrequency ablation, and partial hepatectomy; and (b) poor MRI quality due to motion artifact (**Figure 1**). This study was approved by the Ethics Committee of Henan Provincial People’s Hospital. The requirement to obtain written informed consent was waived due to the retrospective nature of the study.

### 2.2 EOB-MRI protocol

MRI scanning was performed using a 3.0T system (Discover MR750; GE Healthcare, Milwaukee, WI, USA) with an 8-channel phased-array surface coil, with the abdominal MRI scan coil centered at the level of the glabellar process, covering the top of the diaphragm to the lower edge of the liver. The hepatobiliary specific contrast agent (Gd-EOB-DTPA, Primovist; Bayer HealthCare, Berlin, Germany) was injected at a dose of 0.025 mmol/kg via the elbow vein using a high-pressure syringe with a flow rate injection of 1.0 mL/s. The acquisition time for scanning the arterial, portal venous, transitional, and hepatobiliary phases were 20–30 s, 60–90 s, 3–5 min, and 20 min after drug administration, respectively. The MRI parameters for the HBP were as follows: a repetition time (TR) of 4.1 ms, time to echo (TE) of 1.9 ms, matrix size of 320 × 192, field-of-view (FOV) of 360 mm × 288 mm, and layer thickness of 5 mm.

### 2.3 MRI imaging analysis

The MRI data were independently reviewed by two senior diagnostic radiologists, and the results were negotiated to reach a consensus. The qualitative imaging parameters included in the analysis were: (a) HBP tumor hypointensity; (b) non-smooth tumor margin, defined as an irregular margin that had a budding portion at the tumor periphery during HBP; (c) peritumoral hypointensity during HBP, defined as a wedge-shaped or flamelike hypointense area of hepatic parenchyma surrounding the tumor during HBP; The quantitative imaging parameters included in the analysis were: (d) tumor size, which was defined as the maximum diameter of the tumor was measured on the axial HBP; and (e) signal intensity (SI) of the HBP lesion and the liver tissue surrounding the lesion, with each measurement avoiding blood vessels, hemorrhage, and cystic lesions. The maximum lesion level was selected for measurement, and the region of interest (ROI) of the peritumoral liver tissue was selected to be about 200 mm^2^. Peritumoral liver parenchyma was selected within ≤2 cm of the tumor. Finally, each area of interest was measured three times and averaged, and the tumor/peritumoral liver parenchyma signal ratio was calculated.

### 2.4 Radiomics analysis

**Figure 2:**
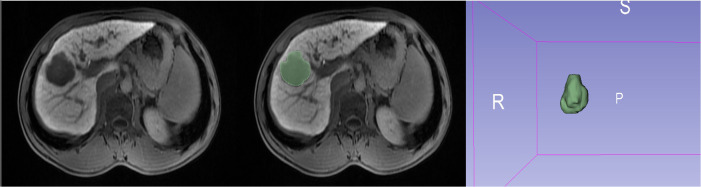
A representative ROI outlined in a 20 min HBP image using 3D Slicer software.

Using an imaging segmentation online software (3D Slicer, version 5.0.3), the ROI was determined by a radiologist with 5 years of abdominal diagnostic experience using the “segment editor” module to manually outline the ROI layer by layer during the HBP, which was later verified and validated by another senior radiologist. If the second radiologist questioned the segmented ROI of the first radiologist, a consensus was reached or the senior radiologist modified it accordingly. After one week, all 137 lesions were outlined again to assess the stability and reproducibility (**Figure 2**). Radiomics profiling was conducted using Python (version 3.9.15) and Pyradiomics (version 3.6.13), after the images were normalized and resampled (1,1,1), resulting in 9 image types (original, wavelet, square, squareroot, logarithm, exponential, gradient, localbinarypattern2D, localbinarypattern3D) and 7 feature classes [shape, first-order statistics, gray-level co-occurrence matrix (glcm), gray-level run length matrix (glrlm), gray-level size zone matrix (glszm), gray-level dependence matrix (gldm), and neighborhood gray-tone difference matrix (ngtdm)].

### 2.5 Selection and construction of the radiomics model

Radiomics features were screened using Python (version 3.9.15). The common features with reproducibility within and between observers were used, while those with intra- and inter-class correlation coefficient (ICCs)<0.75 were excluded. After removing unstable features, t-tests were performed between the positive and negative groups, and least absolute shrinkage and selection operator (LASSO) regression analysis was performed for features with P-values <0.05. The penalty term coefficient (λ) was determined by a 10-fold cross-validation iteration to select the largest AUC. According to the final 11 radiomic features obtained, clinical factors and imaging signs were incorporated using logistic regression (LR) classification to construct a nomogram for comparison. Finally, plots for receiver operating characteristic curves (ROC), calibration curves, and decision curves analysis (DCA) were created to validate the model.

### 2.6 Laboratory tests and histopathological examinations

The preoperative laboratory indicators were serum alpha-fetoprotein (AFP), carcinoembryonic antigen (CEA), carbohydrate antigen 199 (CA199), carbohydrate antigen 125 (CA125), alanine aminotransferase (ALT), aspartate aminotransferase (AST), total bilirubin (TBIL), direct bilirubin (DBIL), and albumin (ALB). In addition, GPC3 immunohistochemistry analysis was reviewed by two pathologists. To accurately assess GPC3 expression, the scoring criteria proposed by Takai et al. (18) was applied, considering the positive cell rate, staining intensity, and staining pattern. Then, the patients were classified into a negative (<10%) and a positive group (≥10%) based on GPC3 expression.

### 2.7 Statistical analysis

Statistical analysis was performed using SPSS Statistics (version 26; IBM Corp, Armonk, NY, USA) and R (version 4.2.1; R Foundation for Statistical Computing, Vienna, Austria) software. The normality of variables was tested using the Kolmogorov-Smirnov test and the homogeneity of variables was tested by Levene’s test. Normally distributed continuous measures are expressed as mean ± standard deviation using the t-test. Non-normally distributed continuous measures are expressed as median (P25–P75) using the Mann-Whitney U test, while the Chi-square test or Fisher’s exact test was used for categorical variables. Inter-reader variability was assessed using Cohen’s kappa coefficient. Clinicoradiologic factors with significant (P<0.05) differences in the univariate analysis were further analyzed using multivariable logistic regression analysis. The area under the curve (AUC), sensitivity, and specificity were used to assess the validity of the predictive model; calibration curve analysis was used to assess the fit between the predicted and actual probabilities of the nomogram model; and the net clinical benefit of the nomogram was evaluated by DCA.

## 3 Results

### 3.1 Patient baseline information

The preoperative clinicopathologic information between GPC3-positive and GPC3-negative patients in both training and validation datasets are shown in **Table 1**. In the training cohort, the two groups demonstrated significant differences in age, serum AFP, non-smooth tumor margins, and tumor/peritumoral liver parenchyma signal ratios. In the validation cohort, two groups demonstrated significant differences in non-smooth tumor margins and tumor/peritumoral liver parenchyma signal ratios.Inter-reader agreement between the two radiologists for HBP imaging was good with a Cohen’s kappa value ranging from 0.761 to 1.000. Univariate logistic regression analysis in the training cohort showed that age, AFP, non-smooth tumor margin, and tumor/peritumoral liver parenchyma signal ratio were significantly associated with GPC3 expression (P<0.05), and all factors were further analyzed using multivariable logistic regression. Age (odd ratio (OR)=0.950; 95% confidence interval (CI): 0.906–0.992, P=0.025) and non-smooth tumor margin (OR=3.388; 95% CI: 1.197–9.649, P=0.020) were found to be independent risk factors for clinicoradiologic modeling, while AFP>400 ng/mL (OR=3.136; 95% CI: 0.950–14.318, P=0.088) possessed clinical significance.

**Table 1:** Comparison of clinical radiological features between GPC3-positive and GPC3-negative patients.

Variable	Training cohort			Validation cohort		
	GPC3-positive (n=103)	GPC3-negative (n=34)	P value	GPC3-positive (n=41)	GPC3-negative (n=8)	P value
Age (years)	54.83 ± 9.88	59.88±8.81	0.009	54.05±1.27	56.63±3.44	0.431
Gender (%)			1.000			0.633
Male Female	84 (81.6) 19 (18.4)	28 (82.4) 6 (17.6)		24 (58.5) 17 (41.5)	6 (75.0) 2 (25.0)	
AFP (%)			0.038			0.333
<400ng/mL >400ng/mL	74 (71.8) 29 (28.2)	31 (91.2) 3 (8.8)		32 (78.0) 9 (22.0)	8 (100.0) 0 (0.0)	
CEA (ng/mL)	2.29 (1.34,3.61)	1.71 (0.96, 2.54)	0.054	1.64 (0.88, 2.16)	2.18 (0.81, 3.13)	0.343
CA199 (U/mL)	19.35 (9.65, 30.75)	14.94 (8.56, 32.06)	0.648	9.43 (4.60, 25.67)	15.46 (12.71, 30.87)	0.310
CA125 (U/mL)	11.61 (7.34, 15.05)	12.05 (9.42, 26.16)	0.212	8.87 (6.21, 16.38)	10.66 (6.79, 16.78)	0.905
TBIL (µmol/L)	13.90 (9.90, 17.70)	14.20 (11.40, 20.90)	0.690	11.90 (8.90, 15.60)	10.85 (8.40, 15.50)	0.735
DBIL (µmol/L)	4.60 (3.25, 6.65)	4.85 (3.23, 6.45)	0.960	3.30 (2.60, 4.50)	2.55 (1.75, 3.68)	0.189
ALT (IU/L)	27.60 (19.05, 42.00)	29.80 (21.70, 41.75)	0.548	31.40 (18.30, 46.80)	22.50 (15.77, 33.65)	0.250
AST (IU/L)	32.00 (23.25, 42.35)	32.55 (24.40, 46.00)	0.519	29.60 (23.00, 43.30)	26.80 (21.12, 32.85)	0.285
ALB (g/L)	40.24 ± 5.35	39.84 ± 4.81	0.695	39.85±4.34	38.50±3.30	0.409
Diameter (mm)	82.7 (67.0-107.0)	83.0 (64.0-103.0)	0.772	32.0 (20.0-49.0)	19.0 (13.0-48.5)	0.185
HBP hypointense			1.000			…
Present Absent	101 (98.1) 2 (1.9)	33 (97.1) 1 (2.9)		41 (100.0) 0 (0.0)	8 (100.0) 0 (0.0)	
Peritumoral hypointense			0.157			0.104
Present Absent	40 (38.8) 63 (61.2)	8 (23.5) 26 (76.5)		21 (51.2) 20 (48.8)	1 (12.5) 7 (87.5)	
Non-smooth tumor margin			0.002			0.037
Present Absent	92 (89.3) 11 (10.7)	22 (64.7) 12 (35.3)		33 (80.5) 8 (19.5)	3 (37.5) 5 (62.5)	
Tumor SI	548.96 ± 156.41	592.62±201.79	0.193	364.07±34.05	402.92±103.01	0.665
Peritumoral SI	875.02 ± 221.85	853.51±199.68	0.616	633.61±49.59	577.28±122.92	0.653
Tumor/peritumoral signal ratio	0.64 ± 0.14	0.71±0.22	0.026	0.55±0.01	0.66±0.05	0.033

GPC3, glypican-3; AFP, alpha-fetoprotein; CEA, carcinoembryonic antigen; CA199, carbohydrate antigen 199; CA125, carbohydrate antigen 125; TBIL, total bilirubin; DBIL, direct bilirubin; ALT, alanine aminotransferase; AST, aspartate aminotransferase; ALB, albumin; SI, signal intensity.

### 3.2 Radiomics feature analysis and radscore calculation

**Figure 3:**
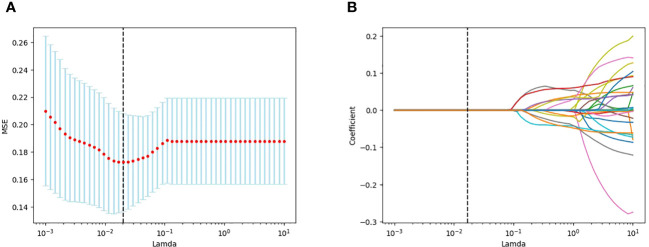
LASSO plots for screening features created using Python. **(A)** Lamda in the horizontal coordinate and mean square error (MSE) in the vertical coordinate. **(B)** Lamda in the horizontal coordinate and coefficients in the vertical coordinate.

**Figure 4:**
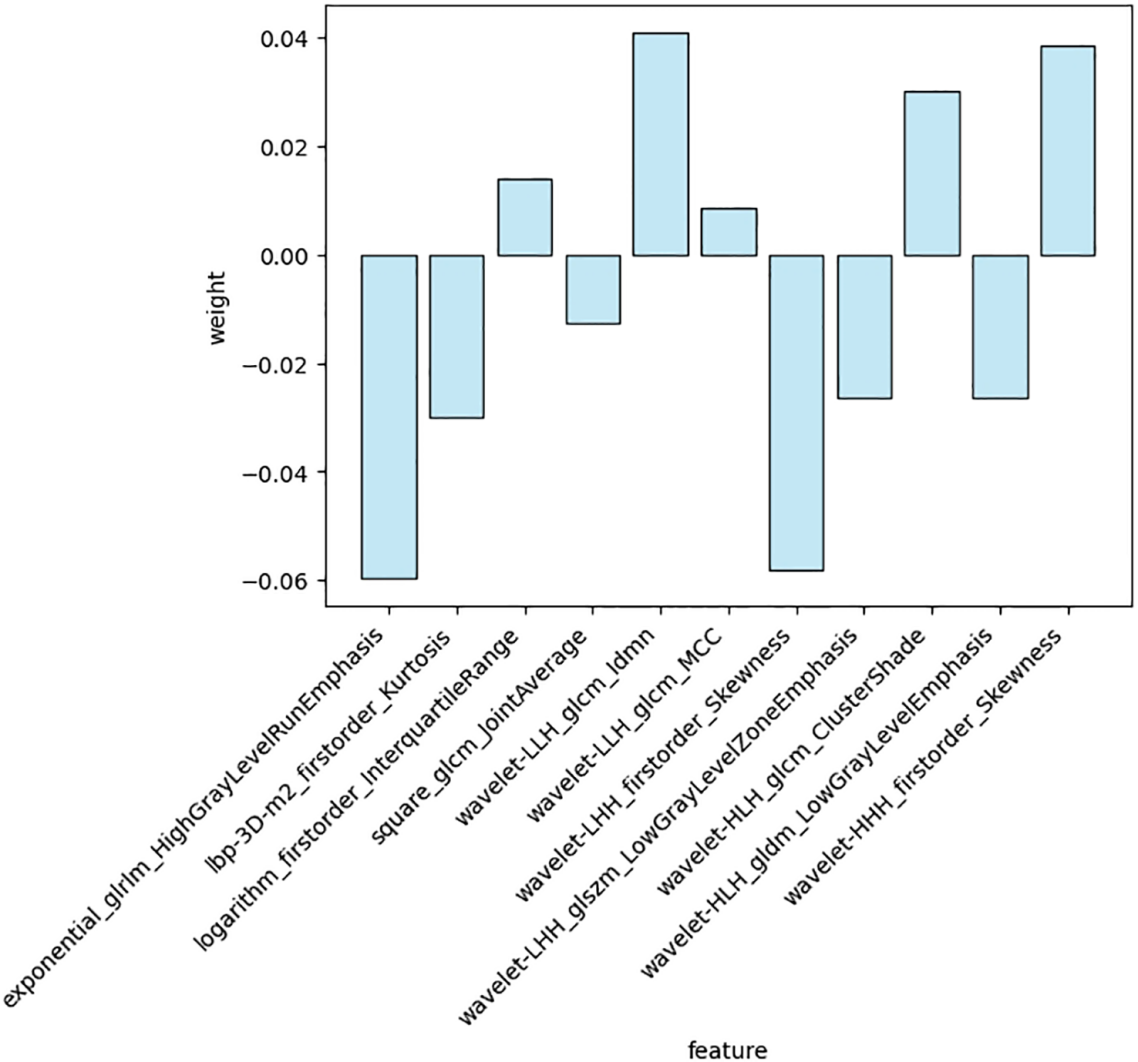
Weighting coefficients of 11 the radiomics features.

A total of 1688 features were extracted from the HBP images per patient in the training cohort, including 14 shape, 18 first-order statistical, 75 texture, 744 wavelet, 93 square, 93 square root, 93 logarithmic, 93 exponential, 93 gradient, and 372 (93 2D and 279 3D) local binary features. Following exclusion of radiomics with ICCs<0.75, 1587 features were judged as stable features and screened using the t-test, retaining 26 features. Of these, 11 key radiomics features were finally identified by LASSO regression analysis (**Figure 3**). The details of the 11 radiomics features are shown in **Figure 4**.

Radscore=(-0.058037)×exponential_glrlm_HighGrayLevelRunEmphasis+(-0.032068)×lbp-3D-m2_firstorder_Kurtosis+(0.015217)×logarithm_firstorder_InterquartileRange+(-0.016434)×square_glcm_JointAverage+(0.041409)×wavelet-LLH_glcm_Idmn+(0.010683)×wavelet-LLH_glcm_MCC+(-0.058619)×wavelet-LHH_firstorder_Skewness+(-0.027467)×wavelet-LHH_glszm_LowGrayLevelZoneEmphasis+(0.032267)×wavelet-HLH_glcm_ClusterShade+(-0.029348)×wavelet-HLH_gldm_LowGrayLevelEmphasis+(0.041051)×wavelet-HHH_firstorder_Skewness.

### 3.3 Modeling and evaluation of radiomics nomogram

**Figure 5:**
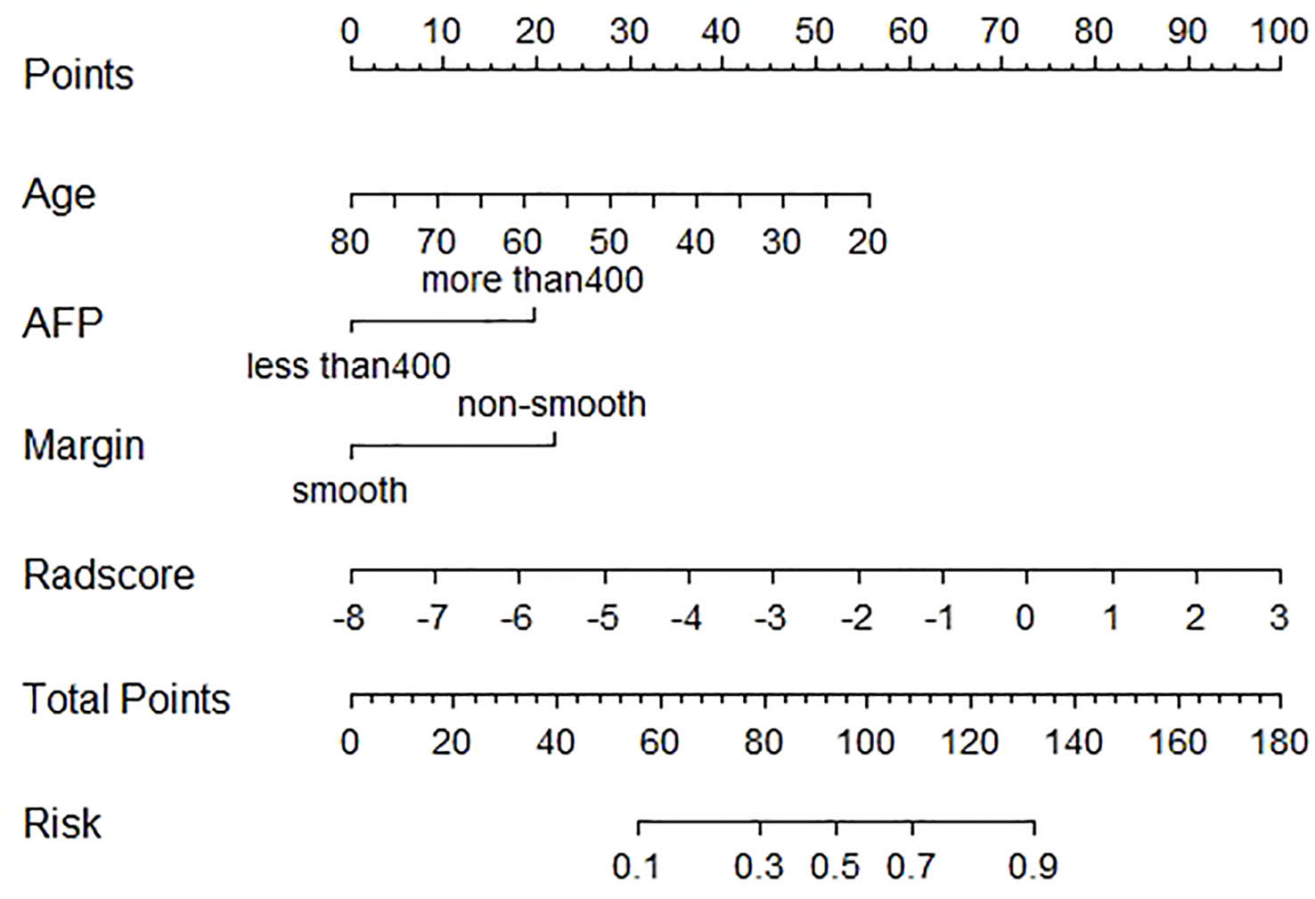
Nomogram of radiomics features and clinicoradiologic features of GPC3 expression in HBP 20min MRI imaging.

**Figure 6:**
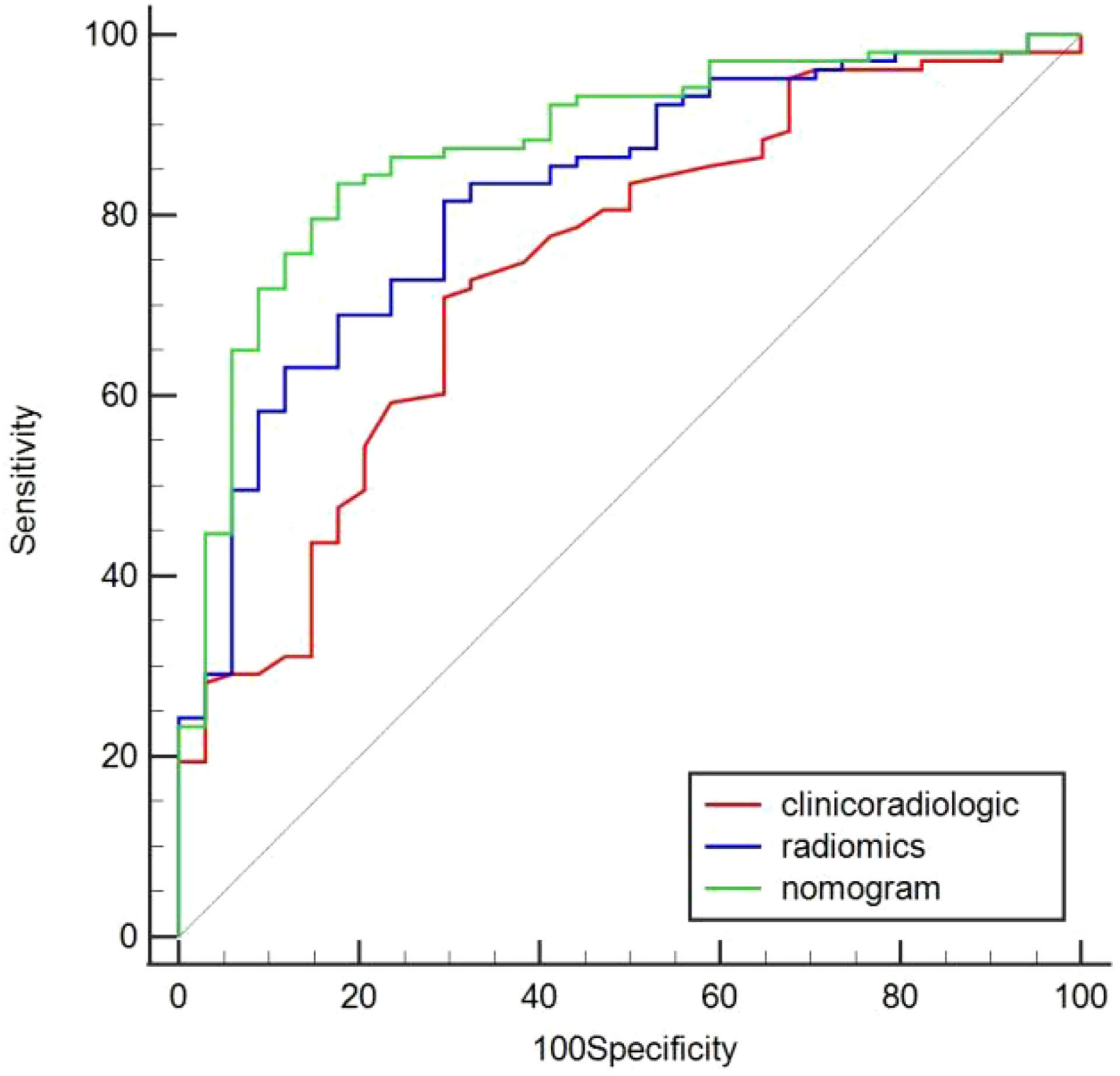
Comparison of predicted GPC3 expression using the area under curve (AUC) for clinicoradiologic, radiomics, and nomogram models in the training cohort.

**Figure 7:**
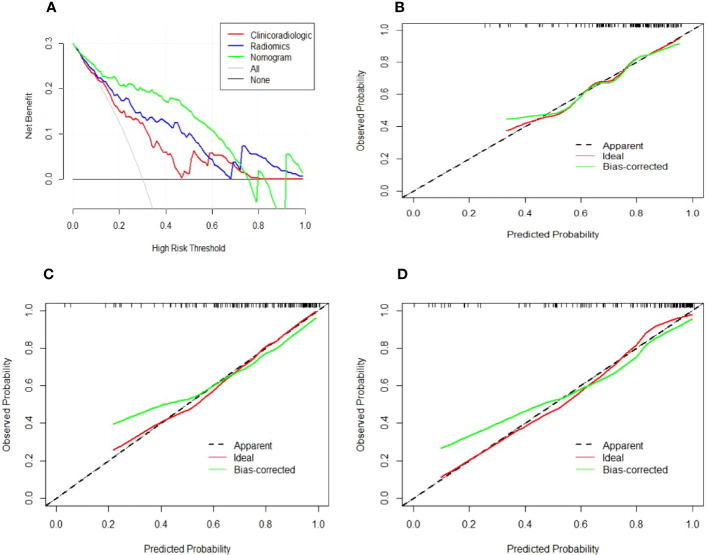
Decision and calibration curves for predicting positive GPC3 expression. **(A)** DCA demonstrating that the nomogram model outperforms the radiomics and clinicoradiologic models for predicting GPC3 in HCC. The gray line indicates the net benefit assuming all patients are GPC3-positive, whereas the black line indicates the net benefit curve assuming all patients are not GPC3-positive. The green, blue, and red lines indicate the **(B)** clinicoradiologic model, **(C)** radiomics model, and **(D)** nomogram model, respectively, with all showing that the predicted GPC3 probability is consistent with the actual probability. The X-axis indicates the predicted GPC3 expression likelihood, while the Y-axis indicates the actual GPC3-positive patients. Moreover, the diagonal dashed line indicates the ideal prediction of the perfect model.

**Figure 8:**
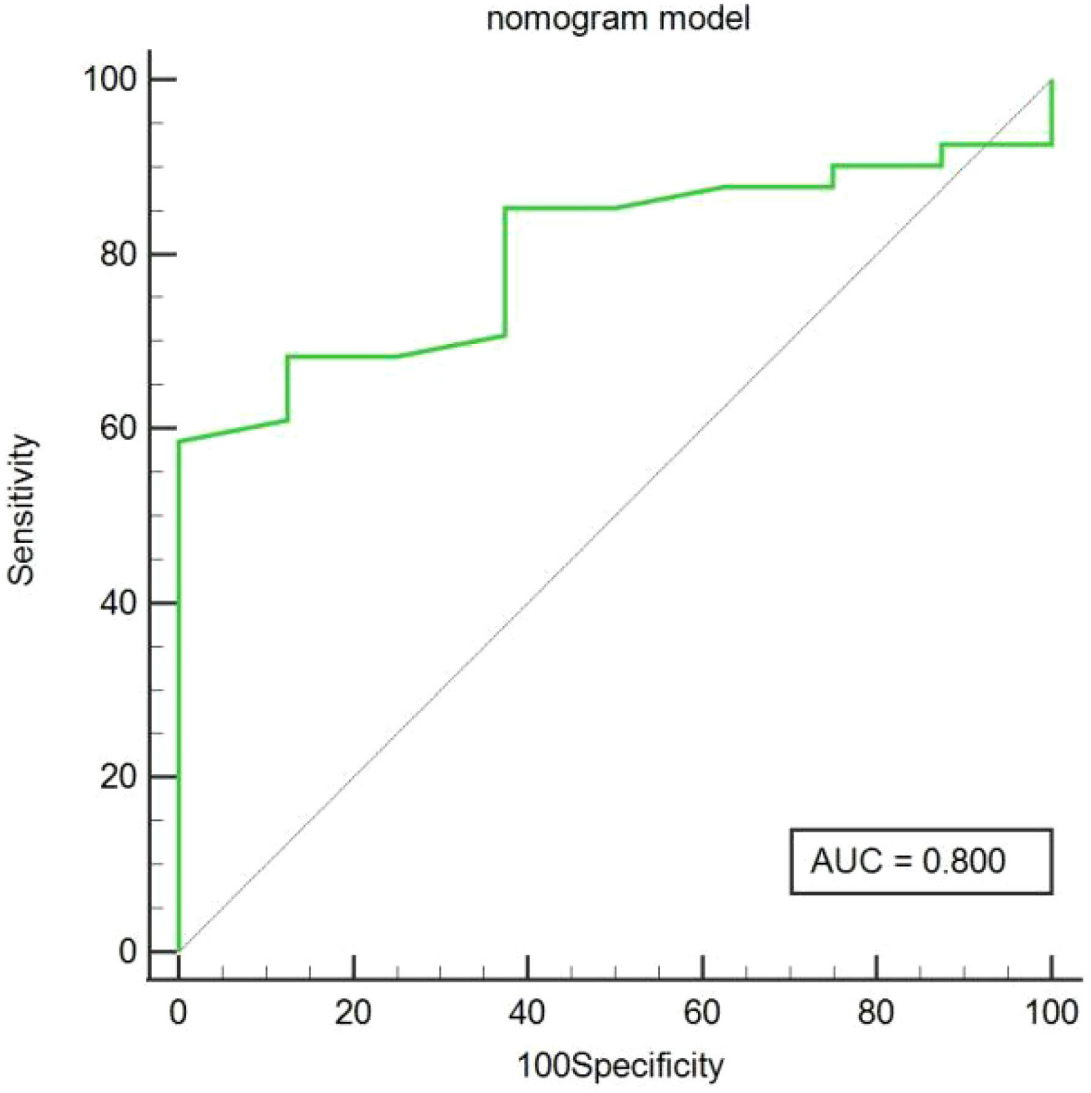
Predicted GPC3 expression using the area under curve (AUC) for nomogram models in the validation cohort.

In the training cohort, age, serum AFP >400 ng/mL, and non-smooth tumor margin were incorporated, and the top 11 features were combined to construct the nomogram (**Figure 5**). The diagnostic performance of each model was evaluated using ROC curves. The clinicoradiologic model had AUC of 0.746, with sensitivity of 76.7% and specificity of 64.7%. The model constructed using the 11 radiomics features had AUC of 0.822, with sensitivity of 81.6%, and specificity of 70.6%. The model consisting of the combined radiomics and clinicoradiologic scores had AUC of 0.888, with sensitivity of 77.7% and specificity of 91.2% (**Figure 6**). The clinical application value of each model was assessed using DCA (**Figure 7A**) and showed that the nomogram yielded a higher net clinical benefit than the radiomic and clinicoradiologic models, indicating the clinical utility for GPC3-positive patients with HCC. The calibration curve assessed the agreement between the actual and predicted GPC3 expression and found close agreement between the predicted GPC3 status of the three models and the actual GPC3 status (**Figures 7B–D**). In the validation cohort, the nomogram model consisting of the combined radiomics and clinicoradiologic scores had AUC of 0.800, with sensitivity of 58.5% and specificity of 100.0% (**Figure 8**).

## 4 Discussion

This study investigated radiomic and clinicoradiologic features during HBP in Gd-EOB-DTPA-enhanced MRI scans of patients with HCC for preoperative prediction of GPC3 expression and constructed a nomogram that achieved good results and yielded a superior predictive performance than the radiomics and clinicoradiologic models. Given the signal difference between tumor tissue and surrounding liver parenchyma in HBP images is more significant than traditional contrast, the radiomics approach facilitates accurate depiction of tumor boundaries.

GPC3 plays a key role in HCC development and tumor cell proliferation and invasion regulation. Most patients with GPC3-positive HCC have a poor postoperative prognosis with early recurrence and a low overall survival rate (6–8). Therefore, early prediction of GPC3 expression using radiomic features is an important clinical tool; however, studies addressing GPC3 radiomics are limited. Although Chong et al. (13) conducted a radiomics study based on Gd-EOB-DTPA-enhanced MRI, the 10 most discriminating radiomic signatures extracted lacked HBP features. In the current study, we extracted HBP features consisting of original, wavelet, square, square root, logarithmic, exponential, gradient, and local binary features. Therefore, more tumor-related signatures can be obtained from HBP images to achieve a quantitative assessment of tumor heterogeneity. Moreover, conventional HBP MRI quantitative parameters, such as tumor signal, peritumor liver parenchymal signal, and tumor/peritumoral liver parenchyma signal ratio, may be a valuable addition to the predictive indexes of existing studies.

Radiomics can transform medical imaging into high-dimensional quantitative imaging feature data, describe tumor heterogeneity more comprehensively and quantitatively, and make up for the lack of qualitative diagnosis in traditional imaging. In our study, 11 features most relevant to GPC3 expression were selected, all of which were high-order features, of which 7 were wavelet features, and the remaining 4 were square, logarithmic, exponential, and local binary features, respectively. These 11 feature parameters reflect, to varying degrees, the differences in HCC GPC3 expression in terms of imaging gray value distribution, texture features, and spatial heterogeneity. Wavelet transformation is a radiomics analysis method with good localization properties that can identify characteristic image features and is more responsive to the internal environment and tumor heterogeneity. In addition, the wavelet transformation can eliminate the noise in images and sharpen images, and the transformed higher-order features can more clearly portray subtle changes in the tumor tissue. Consisted with our findings, Gu et al. (14) identified MRI-derived wavelet features following wavelet filtering, which focused on different frequency ranges: those with high frequency reflected the tumor edge and detailed information, while those with low frequency obtained the tumor outline information and filtered noise at the same time. Furthermore, Qu et al. (19, 20) constructed a radiomics model with mostly wavelet-filtered imaging features to predict microvascular invasion (MVI), indicating that the wavelet filter is a powerful tool for obtaining decomposition and approximation information of imaging. The results of this study show that four radiomics features such as exponential_glrlm_HighGrayLevelRunEmphasis, wavelet-LLH_glcm_Idmn, wavelet-LHH_firstorder_Skewness, wavelet-HHH_firstorder_Skewness have large weights. The screened exponential is based on the glrlm feature, which describes the alignment of pixels with the same gray level in a specified direction. exponential_glrlm_HighGrayLevelRunEmphasis describes the complexity of the GPC3-positive HCC lesion site and the change of layers, which indicates heterogeneity of the lesion structure. The screened wavelet features are mainly based on glcm and first-order statistics, glcm reflects the spatial relationship between pixels and determines the frequency of occurrence of a particular combination of pixels in the image, first-order statistical features mainly describe distribution of pixel or voxel intensities within the tumor region of the image, and Skewness represents the degree of asymmetry of imaging in histogram distribution, which reflects the fact that grayscales of the GPC3-positive HCC are more asymmetric, inhomogeneous, and heterogeneous than those of the negative ones.

In the training cohort, our results showed that patients in the GPC3-positive group were younger than those in the GPC3-negative group, which was consistent with previous reports (13–15). Univariate logistic regression analysis showed AFP levels were significantly different between the groups, with the positive group more likely to have AFP levels >400 ng/mL. However, multivariable analysis showed no significant difference between the two groups. AFP has become the most widely used clinical biomarker for the diagnosis and prognosis of HCC, and high AFP levels have been shown to correlate with HCC progression, postoperative tumor recurrence, and metastasis. Weng et al. (21) reported that GPC3 shared the same pattern of regulation with AFP, whereby a reduction in zinc finger family protein−zinc finger and BTB domain-containing protein 20 (ZBTB20) expression promoted the expression of both AFP and GPC3, as well as hepatocytes proliferation, consistent with findings of previous studies (13–17). In the validation cohort, There was no statistically significant difference in age and AFP between groups, which we speculated may be related to smaller sample sizes. Several studies have demonstrated that a non-smooth tumor margin during HBP is associated with MVI, risk of early recurrence, and poor prognosis (22, 23). In this study, patients with GPC3-positive HCC predominantly showed irregular tumor margins, which may be due to the higher malignancy of GPC3-positive HCC and the susceptibility of tumor marginal tissues to invasion, resulting in changes in morphology (24). Univariate analysis of the tumor/peritumoral liver parenchyma signal ratio revealed a significant difference between the two groups. In addition, evidence suggests that poorly differentiated HCC cells cannot express OATP1B3 on the membrane surface; which contributes to low signal on MRI due to poor contrast uptake (25). Conversely, Gong et al. (26) observed that GPC3 expression was higher in moderately and poorly differentiated HCC than in highly differentiated HCC. Thus, the lack of significant differences in multivariate analysis may be attributable to the interaction of multiple factors.

This study had several limitations. First, this was a single-center retrospective study. In the future, we plan to obtain further data for validation to achieve a better outcome. Second, although the combination of t-test and LASSO regression analysis has high efficiency and sparsity, it can be less stable when a large number of features are included in the model. Therefore, other feature selection methods should be investigated in future research. Lastly, we only used HBP images in this study, however, a variety of sequences, such as T2WI, DWI, PRE, AP, PVP, and TP, should be investigated in multiparametric studies.

In conclusion, HBP radiomic features were closely associated with GPC3-positive expression, and the combination of age, AFP >400 ng/mL, and non-smooth tumor margin provided an effective way to non-invasively and individually predict GPC3-positive HCC patients. Thus, this study provides a novel nomogram with clinical utility and objectivity that may help to determine suitable treatment plans for GPC3-positive patients with HCC in the future.

## Data availability statement

The original contributions presented in the study are included in the article/supplementary material. Further inquiries can be directed to the corresponding author.

## Ethics statement

The studies involving human participants were reviewed and approved by ethics committee of Henan Provincial People’s Hospital. Written informed consent for participation was not required for this study in accordance with the national legislation and the institutional requirements.

## Author contributions

Study design: NZ, MW and SZ. Data collection: YZ, CY, CW, DS, MG and YL. Data analysis: NZ, YZ, and CY. Supervision: SZ. Writing of the original version: NZ. Revision of the paper: MW and SZ. All authors contributed to the article and approved the submitted version.
